# Apicomplexans: A conoid ring unites them all

**DOI:** 10.1371/journal.pbio.3001105

**Published:** 2021-03-11

**Authors:** Julien Guizetti, Friedrich Frischknecht

**Affiliations:** Parasitology, Centre for Infectious Diseases, Heidelberg University Hospital, Heidelberg, Germany

## Abstract

Apicomplexan parasites are defined by complex apical structures, which are necessary for interaction with incredibly diverse host cells. Two studies now amend a long-standing paradigm by showing conservation of an essential ring structure in the entire phylum.

Apicomplexan parasites are eukaryotic protozoan organisms and close relatives to ciliates and dinoflagellates. Four decades ago, Apicomplexa were classified into Conoidasida and Aconoidasida indicating presence or absence of a specific apical cell structure called the conoid that contains several rings. Super-resolution microscopy and proteomic data published in 2 different studies now suggest that this classification might be obsolete [1,2]. Key members of the Aconoidasida include the malaria-causing *Plasmodium* spp., which infect many different vertebrates and have been one of the major human pathogens throughout history, as well as *Theileria*, which can cause cancer in cattle by infecting and transforming cells of the immune system. Conoidasida are represented by *Toxoplasma gondii*, which is the most successful human parasite currently infecting about a third of all people; *Cryptosporidium*, the second leading cause of human diarrhea; *Eimeria* that infects a wide range of vertebrates including farm animals and Gregarines, which are one of the key drivers of biological diversity in neotropical rainforests [3]. Many apicomplexans undergo complex life cycles involving different hosts. They can be transmitted from ticks to cows, from mosquitoes to humans, and from mice to cats, and they can be swallowed or transmitted sexually.

Apicomplexans can change their forms in the most dramatic imaginable ways as they progress along their life cycle; they can grow flagella in one life cycle stage to swim, while in others, they migrate at high speed without any appendages and then replicate within a host cell into tens of thousands of progeny parasites. They compete for the prize of the smallest eukaryotes but can also be nearly a millimeter long. Many apicomplexan species still await discovery, while some of the known ones show remarkably unique and fascinating biology, the molecular bases of which are understudied. Best investigated are the malaria-causing human and rodent-infecting *Plasmodium* species and *T*. *gondii*, which can be grown in tissue culture and mice and genetically manipulated at ease.

The apicomplexans are highly polarized cells that evolved the namesake set of apically located organelles and cytoskeletal structures that underpin their huge success as predators [4]. The apical complex allows the parasites to attach to a host cell and suck out their content or to invade it. This highly specialized front end of the parasite contains a variable number of rings, the composition of which being currently discovered. The apical rings can become smaller the closer they are to the front end of the parasite. From the largest ring microtubules emanate into the cytoplasm toward the nucleus, delineate the circumference of the parasite, and as essential structural components of the pellicle give the parasites their shape [5]. These microtubules encase a set of vesicles that can secrete proteins important for parasite motility and host cell invasion (Fig 1).

**Fig 1:**
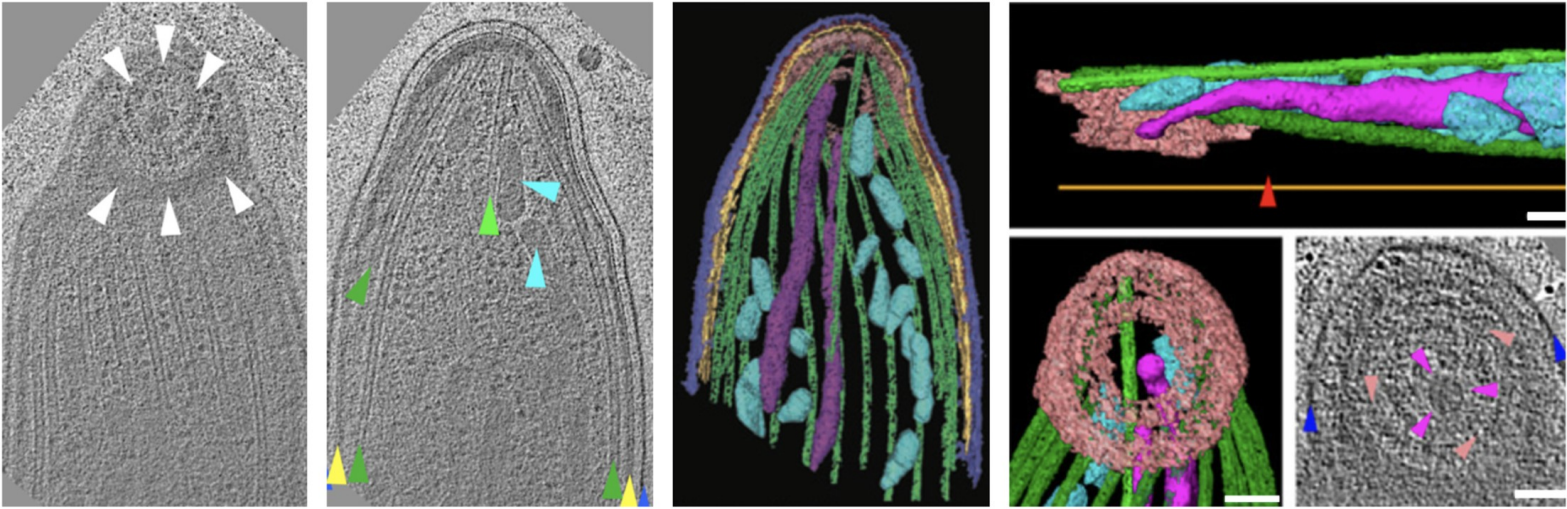
Rings in the apex of an apicomplexan parasite. The front (apical) end of apicomplexan parasites is specialized for secretion of proteins involved in motility and host cell invasion. Secretory vesicles that contain different sets of proteins are encased by microtubules and concentric rings that provide an aperture at the tip for secretion. The numbers and shape of the rings and vesicles vary considerably between parasites. Shown here are selected sections from a tomographic reconstruction of the front end of a *Plasmodium* sporozoite, the form of malaria parasites transmitted by mosquitos. *Plasmodium* sporozoites are unusual as they show apical complex rings reoriented so that they are not perpendicular to the cell longitudinal axis, but face the substrate. The colored images represent 3D models derived from the electron microscopy data. Visible structures and organelles are indicated by arrowheads with matching color in the models unless otherwise stated. White and pink arrowheads indicate the apical rings, which are colored in pink; red arrowhead indicates a sporozoite specific kink, green: microtubules, blue: plasma membrane, yellow: the inner membrane complex (alveoli) that defines alveolates, cyan and magenta: vesicles releasing proteins involved in migration (micronemes, cyan) and invasion (rhoptries, magenta). Scale bars: 100 nm. Images from [10].

True to proud parasites, apicomplexans aren’t subtle in going about their business. Some, like *T*. *gondii*, can invade literally any nucleated cell that is large enough to harbor them. Others, like the tiny *Plasmodium* merozoites, invade only red blood cells but grow to unimaginable numbers within their hosts; a human might harbor more than half a kilogram of circulating *Plasmodium* parasites. As invasion of host cells is key to the success of these parasites, the understanding of its molecular details, fascinating per se, might lead to new ways of treating or preventing the diseases caused by them. Many questions can be asked: (i) how is the apical end of these parasites organized; (ii) what are the different structures made of; (iii) how do the different structures interact with each other; (iv) what changes occur during migration or upon host cell binding; (v) which proteins do the parasites secrete and how; and (vi) how is secretion, motility, and invasion regulated? Two papers in this issue of *PLOS Biology* contribute toward answering the first 3 questions.

A recent breakthrough technology in cell biology was the development of ultrastructure expansion microcopy (U-ExM) [6]. By denaturing cells in a gel and subsequent swelling of the gel, those cells could be expanded several fold, while the relative positions of their components were preserved. Consequently, 3D super-resolution imaging can be achieved on classical confocal microscopes. This clearly exciting technique for general cell biologists studying large tissue culture cells is simply a game changer for those studying small protozoans. Studies in *T*. *gondii* already showed the power of U-ExM revealing microtubule twisting during parasite invasion [7] and microtubule disruption by the absence of proteins essential for apical ring formation [8]. Now, Bertiaux and colleagues [1] use U-ExM for the first time in 2 species of *Plasmodium* and reveal a number of interesting insights into the arrangement of the microtubules in different stages of the parasite. Most striking is the discovery of a tubulin-based ring at the tip of the ookinetes, the motile forms of the parasite that penetrate the mosquito gut. Using co-labeling with known proteins, they could also show that the ring is moving as ookinetes are activated to migrate. U-ExM, however, did not clarify whether the ring is made from a tubulin polymer. If this were the case, it would be an incredible discovery, considering the sharp curvature the polymers would have to take. This could be investigated in the future, possibly by electron tomography. The movement of the ring is strikingly similar to a shifting structure that is found in *T*. *gondii*. In this parasite, not just a ring moves but a highly curved barrel-shaped array of tubulin polymers known as the conoid [4]. This tubulin polymer is so tightly curved that the tubules don’t close and hence are not considered bona fide microtubules. In resting *T*. *gondii*, the conoid sits below the large ring in the cytosol, and upon activation, it is moved through this ring. Malaria parasites, along with some other Aconoidasida, were previously believed to lack such a conoid. However, the study by Koreny and colleagues now identifies an important number of novel proteins that are located at the conoid in *T*. *gondii* and demonstrates their presence at the apical end of different *Plasmodium* stages [2]. To achieve this, the authors mix their recently established spatial proteomics method (hyperLOPIT) based on subcellular fractionation and mass spectrometry [9] with proximity ligation, a method that allows the identification of proteins in the near neighborhood of a tagged protein. Strikingly, all such identified proteins did localize to the front end of the parasites, a rare feat considering that such methods frequently identify false positives. Some of the identified proteins were found in a ring at the tip of malaria parasites by super-resolution microscopy. Together with their extended orthology analysis in related taxa, this suggests that the apical ends of Apicomplexa show a higher degree of molecular conservation than previously thought. This is particularly interesting as some species developed unique conoid-like structures, which can now be investigated in more detail. The combined insights and technical advances reported in these 2 papers will enable a deeper and functional look into the molecular makeup and organization, not only of the apical end of these parasites, but also into many other fascinating aspects of the unique biology of Apicomplexa.
